# VEGF-C serum level is associated with response to bevacizumab maintenance therapy in primary ovarian cancer patients

**DOI:** 10.1371/journal.pone.0269680

**Published:** 2022-06-10

**Authors:** Yi Ding, Leticia Oliveira-Ferrer, Eike Vettorazzi, Karen Legler, Karin Milde-Langosch, Linn Woelber, Anna Jaeger, Katharina Prieske, Volkmar Mueller, Barbara Schmalfeldt, Sascha Kuerti

**Affiliations:** 1 Department of Gynaecology and Gynaecologic Oncology, University Medical Centre Hamburg-Eppendorf, Hamburg, Germany; 2 Department of Medical Biometry and Epidemiology, University Medical Center Hamburg-Eppendorf, Hamburg, Germany; Ottawa Hospital Research Institute, CANADA

## Abstract

**Objective:**

At present, maintenance therapy with the antiangiogenic agent bevacizumab or with PARP-inhibitors represent two options for BRCA-wildtype ovarian cancer patients, after platinum-based first line chemotherapy. The identification of molecular markers to predict patient response to different maintenance therapies remains a major challenge. In the present study we analyzed the predictive potential of vascular endothelial growth factor C (VEGF-C) to identify ovarian cancer patients that might benefit from an antiangiogenic therapy.

**Methods:**

101 patients with primary epithelial ovarian cancer were analyzed for serum levels of VEGF-A,–C and CA-125 by ELISA. Serum levels were compared between patients with low pT-stage (pT1a-pT2c n = 11), healthy individuals (n = 27) and patients with higher pT-stage (> = pT3 n = 90). Adjusted ROC curves and an adjusted logistic regression model were carried out to evaluate the potential impact of VEGF-A and -C, as well as CA-125 serum level concentration on bevacizumab-therapy response, under consideration of covariates such as FIGO, pM, pN and residual tumor after surgery.

**Results:**

A patient which has in comparison twice the VEGF-C concentration in serum, has a significant increased chance of response to bevacizumab by a factor of 2.79. Further, only VEGF-C serum levels were significantly higher in the group of patients with lower pT-stage compared to healthy individuals, whereas VEGF-A or CA-125 serum levels could not discriminate between healthy individuals and patients with ovarian cancer at low pT-stages.

**Conclusion:**

VEGF-C serum level might serve as as a biomarker to evaluate treatment response under bevacizumab.

## Introduction

Epithelial ovarian cancer (EOC) represents the leading cause of death in gynaecological malignancies [1]. After cytoreductive surgery, patients underwent a first line platinum based chemotherapy. At present, there is the possibility of antiangiogenic first line maintenance therapy with bevacizumab or first line maintenance therapy with the PARP-inhibitor olaparib or niraparib, depending of the BRCA mutation status.

The growth of the EOC tumour cells is dependent on vascular endothelial growth factor (VEGF) mediated angiogenesis [2]. The VEGF family consists of VEGF-A,-C,-D and induces different cascades via their receptor-tyrosine-kinases VEGFR-1, VEGFR-2, VEGFR-3 in order to exert their various biological effects [3].

Bevacizumab, a recombinant humanized monoclonal antibody (mAb) has been shown to effectively block VEGF-A, thereby inhibiting tumor-induced angiogenesis [4]. Some patients showed prolonged remission under bevacizumab, while some patients showed rapid recurrence [5, 6].

As described above, several pathways promote tumour growth within the VEGF family. VEGF-C has been demonstrated as a potent enhancer of tumour lymphangiogenesis, leading to increased metastatic spread of tumor cells to lymph nodes but also to distant organs in different entities [7]. Besides, a potential role of VEGF-C as an alternative pro-angiogenic factor has been described. In glioma cells, bevacizumab induces expression of VEGF-C and -D, suggesting a possible escape mechanism from anti-VEGF therapy [8].

Regarding the actual results for the primary PARP-Inhibitor therapy with olaparib after standard chemotherapy, a molecular marker to determine the additional effect of bevacizumab could be useful in patients with confirmed BRCA mutation. In the PRIMA trial patients with newly diagnosed advanced ovarian cancer who had a response to platinum-based chemotherapy, those who received niraparib had significantly longer progression-free survival than those who received placebo, regardless of the presence or absence of homologous-recombination deficiency [9]. Furthermore, in those patients without BRCA mutation, a molecular marker to evaluate a possible response of antiangiogenic therapy with bevacizumab is needed.

The aim of the present study was to identify the predictive potential of VEGF-C for the identification of ovarian cancer patients being at risk for progression under bevacizumab therapy.

## Material and methods

### Patients

A total of 101 patients with primary EOC treated at the University Medical Centre Hamburg-Eppendorf between 2009 and 2018 were included in the study. All patients underwent primary debulking surgery according to current German guidelines, as well as standard chemotherapy and bevacizumab treatment [10]. Patient cohorts were classified according to their tumour stage.

To ascertain a difference in the serum expression of VEGF-C in patients with and without progression under bevacizumab therapy, the progression cut off was defined with 18 months treatment. The cut off was defined according to the progression free survival (PFS) of 18.1 months of patients with bevacizumab maintenance therapy compared to the PFS of 14.5 months of patients without bevacizumab maintenance therapy after platinum based chemotherapy, performed in the ICON7 trial [11]. Patients with progression under bevacizumab therapy were defined as non-responder. Patients with progression after 18 months bevacizumab maintenance therapy were defined as responder.

In addition, the cohort is divided into patients with ovarian cancer with low pT-stage (until pT2c, n = 11) and patients with ovarian cancer with high pT-stage (> = pT3, n = 90), independent from nodal status and FIGO stage. Although patients with pT2c pN1 belong to FIGO stage IIIA1, we assume that patients with lower pT stage and lymphnode involvement have a different tumour differentiation than patients with pT3C stage (FIGO IIIC) [12]. Both subgroups of different pT-stages were compared with a group of healthy individuals, which have no diseases (n = 27).

Detailed patient characteristics are presented in Table 1. All patients gave written informed consent to access their tissue and review their clinical records according to our investigational review board and ethics committee guidelines (#200814). The ethics committee of the medical association Hamburg approved the general use of this tissue for retrospective investigations, including this study. Clinical data was retrieved from a detailed database providing information on clinicopathologic factors, histologic classifications and therapeutic procedures. Clinical outcome of all patients was followed from date of first diagnosis until the end of 2018.

**Table 1:** Patient characteristics (n = 128).

	healthy	low pT-stage	high pT-stage
**No. of patients**	27	11	90
**Age at diagnosis (years)**			
Median	60	63,5	62
Range	28–71	54–73	26–79
**Tumour stage**			
pT1b	0	1	0
pT1c	0	1	0
pT2a	0	2	0
pT2b	0	3	0
pT2c	0	4	0
pT3a	0	0	3
pT3b	0	0	14
pT3c	0	0	73
**Lymph node status**			
N0	0	2	19
N1	0	8	60
NX	0	1	11
**Grading**			
G1	0	1	4
G2	0	2	5
G3	0	8	79
Not determined/unknown	0	1	2
**Histologic subtype**			
Serous	0	9	82
Endometrioid	0	2	8

### Serum samples

The blood from primary EOC patients at the time point of surgery and healthy individuals were collected before treatment in monovette serum-gel 7.5 ml blood collection systems (Sarstedt, Germany), then centrifuge at the speed of 2500 g for 20 minutes. The supernatant, serum, was aliquoted into 2 ml tubes and stored in -80°C fridge. All serum samples stayed on the ice until fully melting before measurement. For VEGF-C and CA-125 measurement, serum samples from ovarian cancer patients and healthy individuals were all diluted 5-fold with the dilution solution provided by the kit. There was no dilution of serum for the VEGF-A measurement.

### ELISA analysis

Serum VEGF-A, -C and CA-125 were measured with the Human VEGF-A, -C and CA-125 DuoSet ELISA kits (R&D Systems, Minneapolis, Minn), respectively. Reagents were freshly prepared to working concentrations before use. All reagents were brought to room temperature before use and were allowed to sit 15 minutes with gentle agitation after initial reconstitution. DuoSet ELISA Ancillary Reagent Kit 2 (R&D Systems, Minneapolis) provides a comprehensive collection of reagents and 96-well plates for ELISA assays. Preparation and procedure were performed as described in the manufacturer’s instructions. Briefly, a 96-well Clear Polystyrene Microplate was coated with VEGF-A,-C or CA-125 Capture Antibody diluted with ELISA Plate-coating Buffer. The plate was sealed and incubated overnight at room temperature. The reagent from each well was removed and washed by filling 400 μL Wash Buffer with a multichannel pipettes. This process was repeated two times for a total of three washes. After each wash any remaining Wash Buffer was removed by inverting the plate and blotting it against clean paper towels. Block plates were added with 300 μL of Reagent Diluent to each well and incubated at room temperature for 1.5 hours. The remove/wash was repeated as before. Subsequently, 100 μL of diluted or undiluted samples, controls and standards were added into each well and incubated 2 hours at room temperature. After several remove/wash steps, 100 μL of the VEGF-A, -C or CA-125 Detection Antibody reagent were added to each well and incubated 2 hours at room temperature. The remove/wash was repeated and 100 μL of the working dilution of Streptavidin-HRP were added to each well. After an incubation time of 20 minutes at room temperature and several wash steps, 100 μL of Substrate Solution were added to each well and incubated for 20 minutes at room temperature avoiding direct light. Finally, 50 μL of Stop Solution were added to each well and the optical density of each well was determined immediately, using a microplate absorption reader (SunriseTM, Tecan Trading AG, Switzerland) at a 450 nm measurement wavelength and a 540 nm reference wavelength.

### Statistical analysis

Values representing VEGF-A, -C and CA-125 expression levels were tested among the different groups by ANOVA analysis, as well as post hoc with the Bonferroni or the Games-Howell test. ROC curves adjusted to covariates (FIGO, pM, pN and tumor rest after surgery) and a logistic regression model with the therapy response/non-response status as outcome, and VEGF-C serum levels adjusted by aforementioned covariates was carried out.

P values <0.05 were considered to be statistically significant. Boxplots were diagrammed on the basis of absolute VEGF expression levels. All statistical analyses were carried out with SPSS (IBM SPSS Statistics version 25 for windows).

## Results

### VEGF-C serum concentration is significantly associated increased response to bevacizumab therapy

**Fig 1:**
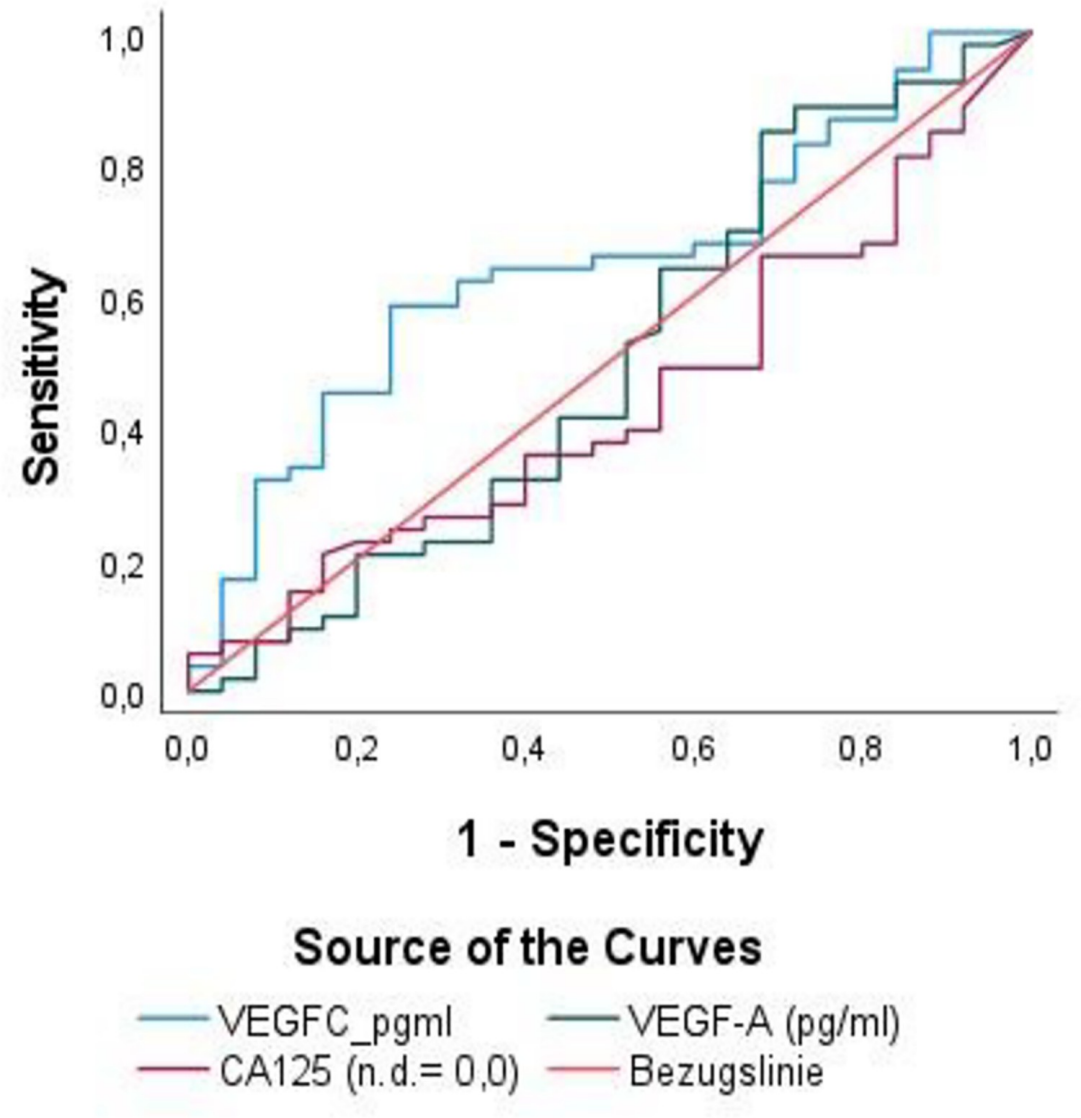
ROC curves for VEGF-C, VEGF-A and CA125 serum levels as markers for becacizumab therapy response.

Serum samples of all included 101 ovarian cancer patients were analysed for CA-125, VEGF-A and VEGF-C expression by Elisa. Since all patients > = FIGO IIIA1 included in this study were treated with bevacizumab as maintenance therapy, we have tested whether these two members of the VEGF-family, VEGF-A and VEGF-C, might show a predictive role for antiangiogenic therapy response. In this context, ROC curves for VEGF-A, VEGF-C and CA-125 adjusted to covariates (pN, pM, FIGO and tumor rest after surgery) were performed in a cohort of 87 patients with the corresponding available information and are shown in Fig 1. Here, the area under the curve (AUC) value for VEGF-C was rather poor (AUC: 64.5%; 95%CI: 0.52–0.78; p = 0.28), and failed for VEGF-A (AUC: 50%; 95%CI: 0.35–0.65; p = 1.0) and CA-125 (AUC:44%; 95%CI: 0.31–058; p = 0.4). However, logistic regression analysis adjusted with the aforementioned covariates revealed a significant association between VEGF-C levels and bevacizumab response (p = 0.034, Exp(B) = 2.79), indicanting that in a patient with twice the VEGF-C concentration in serum, the chance of response to bevacizumab therapy increases by a factor of 2.79. In contrast, no significant association between VEGF-A or CA-125 serum values and bevacizumab therapy response was found.

### Ovarian cancer patients with low pT-stage exhibit significant higher serum levels of VEGF-C compared to healthy individuals

**Fig 2:**
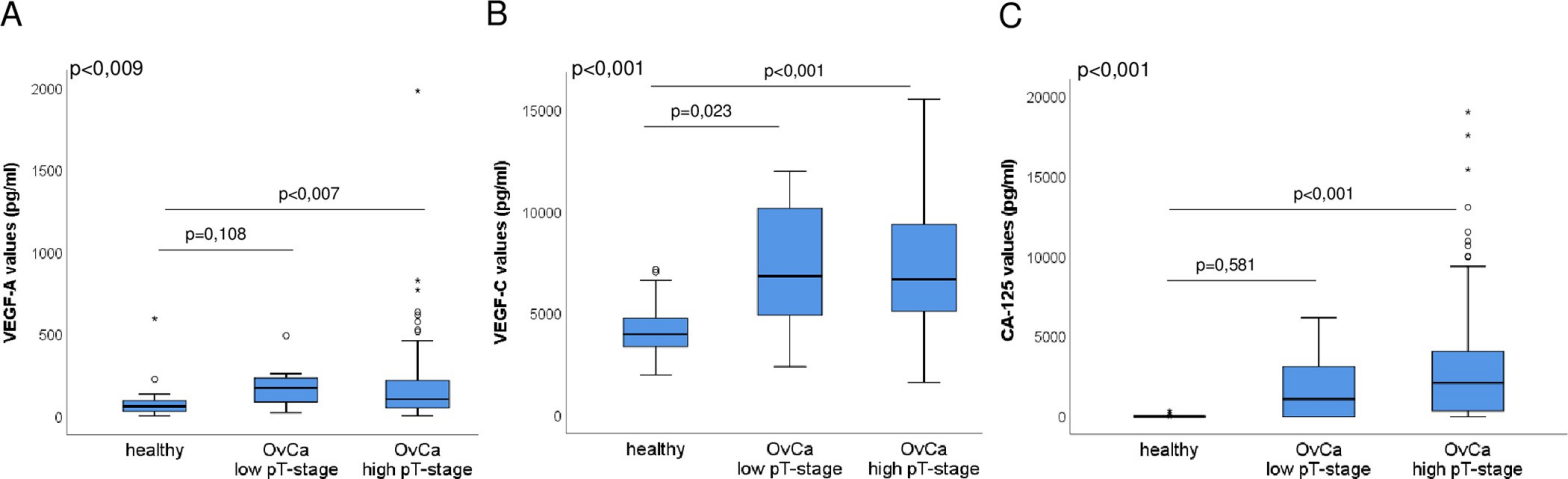
VEGF-A, -C and CA-125 serum levels in healthy and ovarian cancer patients. Box plots showing serum levels of VEGF-A (A), VEGF-C (B) and CA-125 (C) in healthy people and ovarian cancer patients with low and high stages. Two types of outliers: out values and extreme (far out) values in box plots were marked with small circle and star respectively.

Additionally, VEGF-A,-C and CA-125 serum levels were measured in healthy individuals and further compared with those of ovarian cancer patients. Here, in comparison to healthy individuals significantly higher serum VEGF-A levels were found in ovarian cancer patients with high pT-stage (mean values 82.78 pg/mL vs 191.97 pg/mL, p<0.007, Fig 2A), but not in ovarian cancer patients with low pT-stage (mean values 82.78 pg/mL vs 180.19 pg/mL, p = 0.108, Fig 2A). In the ovarian cancer case cohort, the high serum VEGF-A levels correlated significantly with high FIGO stage (p = 0.008, Table 2). However, the serum VEGF-A levels were not significantly associated to other clinico-pathologic parameters (Table 2).

**Table 2:** Correlation between clinico-pathological parameters and serum VEGF-A, VEGF-C and CA-125 levels in the ovarian cancer cohort.

		VEGF-A *	p-value	VEGF-C *	p-value	CA-125*	p-value
**Stage**	low pT stage (pT1-2c)	180,19	0,884	7361,09	0,903	1717,68	0,192
	high pT stage (> = pT3)	191,97		7246,02		3440,47	
**FIGO**	I	84,88	**0,008**	7711,18	0,844	3235,68	0,976
	II	174,26		7278,78		3131,05	
	III	310,75		7057,26		3185,53	
**Grading**	G1	129,24	0,83	8279,8	0,672	412,86	0,244
	G2	176,51		6750,86		2818,88	
	G3	197,77		7297,33		3458,63	
**pN**	0	119,91	0,099	6508,29	0,231	2384,95	0,339
	1	228,3		7408,57		3351,98	
**pM**	0	162,5	0,186	7302,94	0,848	3304,29	0,925
	1	309,08		7156,37		3205,16	
**OP tumour rest**	not macroscopically visible	202,85	0,602	7340,57	0,55	2558,76	**0,041**
	≤ 1cm^3^	205,66		7621,3		5186,52	
	> 1cm^3^	137,95		7251,94		3846,24	

* mean values (pg/ml)

No significant difference in the CA-125 levels could be found between healthy individuals and ovarian cancer patients with low pT-stage (p = 0.581, Fig 2C). As expected, the CA-125 serum levels in ovarian cancer patients with high pT-stage is significantly higher (p<0.001, Fig 2C) compared to healthy people. A significant correlation between the high CA-125 serum levels and macroscopically visible residual tumour was observed (p = 0,041, Table 2).

Interestingly, compared to the healthy individuals, significantly higher serum VEGF-C levels could be detected not only in ovarian cancer patients with high pT-stage (mean values 4258.89 pg/mL vs 7236.02 pg/mL, p<0.001, Fig 2B), but also in ovarian cancer patients with low pT-stage (mean values 4258.89 pg/mL vs 7361.09 pg/mL, p = 0.023, Fig 2B). However, no significant correlation was found between the VEGF-C serum levels and stage or other clinicopathologic features in ovarian cancer patients (Table 2).

## Discussion

The identification of patients with ovarian cancer in early tumour stage is an important challenge of the current research in ovarian cancer. The tumour marker CA-125 is a commonly used molecular marker to estimate the progression of ovarian cancer patients during therapy. However, CA-125 levels do not reliably discriminate between patients with ovarian cancer in early tumour stage and healthy individuals. Although our subgroup of low (< = FIGO IIC and FIGO IIIA1) and high pT-stage (FIGO IIIC) cannot reliably differ between patients in early (FIGO < = IIc) and late tumour stage (FIGO > = IIIC), our results indicate a different expression of the investigated serum-marker in different pT-stage.

While there were significantly increased CA-125 values in serum samples of patients with high pT-stage ovarian cancer compared to healthy individuals, there is no significant difference in the CA-125 levels between healthy individuals and ovarian cancer patients with low pT-stage in our study. In contrast, we could show a significant higher expression of VEGF-C serum levels in ovarian cancer patients with low pT-stage compared to healthy individuals, without previous screening for familial/genetic ovarian cancer risk. For screened women at familial/genetic ovarian cancer risk, Skates et al. indicate, that ROCA q3 months (risk of ovarian cancer algorithm) had better early-stage sensitivity at high specificity, and low yet possibly acceptable positive predictive value compared with CA-125 > 35 U/mL q6/q12 months [13]. Our findings indicate, as Skates described, that CA-125 alone has not the potential for a screening marker for detection of patients with early ovarian cancer. Further studies with reliable differentiation between low and high FIGO tumour stage is needed to verify a possible increased VEGF-C serum level of patients in early ovarian tumour stage.

Bevacizumab is a humanized VEGF-A binding antibody, which is used to inhibit angiogenesis and tumour growth in patients with advanced tumour stage in addition to chemotherapy. While some patients have a prolonged response under maintenance with bevacizumab, the majority shows a progression. Other than anticipated, we did not find a significant difference in VEGF-A levels at diagnosis between patients showing recurrence disease and those without progression under platinum plus maintenance bevacizumab therapy. Similarly, no significant difference in the CA-125 levels at the time point of surgery was observed between the aforementioned patient subgroups. In contrast, we found a significant association between the VEGF-C serum level and patient response to therapy. Here, a twice VEGF-C serum level increase at the time point of surgery improved the probability of response during bevacizumab therapy by a factor of 2,79. This finding, showing a potential favorable predictive role of pre-treatment VEGF-C levels, but not VEGF-A levels for bevacizumab response, is unexpected concerning a possible escape mechanism from anti-VEGF therapy [8].

Kommoss et al. identified that ovarian carcinoma molecular subtypes with the poorest survival (proliferative and mesenchymal) derive a comparably greater benefit from treatment, including bevacizumab, than the other subtypes [14]. In previous studies from our group, we attributed ovarian tumours with high tumoral VEGF-C expression, measured by Western blot, a mesenchymal tumour type, which is associated with larger tumour conglomerates, in comparison with those ovarian tumorsdisplaying small miliar peritoneal dissemination, which were assigned an epithelial phenotype [12, 15]. Mesenchymal tumour masses are more dependent of neovascularization, which could be an explanation for the better response of antiangiogenic maintenance therapy in patients with high VEGF-C serum levels. In this context, VEGF-C serum levels might represent an usefull and additional parameter for the classification of ovarian tumors into the mesenchymal-like subtype, and in turn to identify patients that might profit from a anti-angiogenic therapy.

Based on the newest results of the SOLO study, patients with detected BRCA mutation profit from the therapy with the PARP inhibitor olaparib as maintenance therapy. In this context, patients with advanced ovarian cancer, detected BRCA mutation and increased serum values of VEGF-C could benefit from simultaneous therapy with bevacizumab and olaparib after primary surgery and first-line chemotherapy. Further, the PRIMA trial showed that niraparib first line maintenance therapy lead to a significantly longer progression-free survival compared to placebo, regardless of the presence or absence of homologous-recombination deficiency [9]. In this context, those patients with BRCA wildtype and increased serum values of VEGF-C could rather benefit from an antiangiogenic therapy with bevacizumab than patients with low serum values of VEGF-C. Whereas patients with BRCA wildtype and decreased serum values of VEGF-C could rather benefit from niraparib first line maintenance therapy instead of bevacizumab.

In conclusion, our findings suggest that the VEGF-C serum level represent a novel biomarker that predicts response to bevacizumab mantinance treatment in primary ovarian cancer patients. Further prospective studys are needed to confirm VEGF-C as a potential useful biomarker to identify patients that might benefit from an antiangiogenic therapy.
